# Complete Genome Sequence of Multidrug-Resistant *Streptococcus pneumoniae* Serotype 19F Isolated from an Invasive Infection in Sapporo, Japan

**DOI:** 10.1128/genomeA.01239-17

**Published:** 2017-11-02

**Authors:** Toyotaka Sato, Yasuo Ohkoshi, Takayuki Wada, Yukari Fukushima, Hiromi Murabayashi, Yasunari Takakuwa, Kaoru Nishiyama, Tsukasa Shiraishi, Chie Nakajima, Yasuhiko Suzuki, Shin-ichi Yokota

**Affiliations:** a Department of Microbiology, Sapporo Medical University School of Medicine, Sapporo, Japan; b Department of Clinical Laboratory, NTT East Sapporo Hospital, Sapporo, Japan; c Department of International Health, Institute of Tropical Medicine, Nagasaki University, Nagasaki, Japan; d Division of Bioresources, Hokkaido University Research Center for Zoonosis Control, Sapporo, Japan; e Department of Respiratory Medicine, NTT East Sapporo Hospital, Sapporo, Japan; f Global Station for Zoonosis Control, Global Institution for Collaborative Research and Education, GI-CoRE), Hokkaido University, Sapporo, Japan

## Abstract

Invasive infection of multidrug-resistant *Streptococcus pneumoniae* is a serious clinical concern. Here, we report the complete genome sequence of a multidrug-resistant *S. pneumoniae* serotype 19F strain isolated from a patient with an invasive infection in Sapporo, Japan.

## GENOME ANNOUNCEMENT

*Streptococcus pneumoniae* is a Gram-positive bacterium that causes respiratory infection, which is responsible for the majority of community-acquired pneumonia in elderly people and acute otitis media in children. Sometimes it causes invasive infections, such as meningitis and sepsis (1, 2). Appropriate and immediate antimicrobial treatment required for the invasive pathogens can be challenging if multidrug resistance is encountered (3, 4). Capsular serotype 19F is the third most common serotype isolated from adult patients with community-acquired pneumonia in Japan (5).

*S. pneumoniae* MDRSPN001, which is serotype 19F and sequence type 10017, was isolated from the blood culture of a 58-year-old female with pneumonia in a hospital in Sapporo, Japan, in 2016. The patient died on the eighth day after onset with occurring disseminated intravascular coagulation. This strain exhibited multidrug resistance. To facilitate further investigation of the genetic basis of the invasiveness and multidrug-resistance mechanism of *S. pneumoniae*, the genome sequence of MDSPN001 was determined using next-generation sequencing technology.

Genomic DNA was isolated from cells of *S. pneumoniae* MDRSPN001 using the Wizard genomic DNA purification kit (Promega, Madison, WI). The complete genome sequence was determined using PacBio RS SMRT Portal (Pacific Biosciences, Menlo Park, CA), and a 1,792.8-Mb sequence was obtained with 90,644 reads and an *N*_50_ read length of 35,986 bp. The average coverage was 701-fold. Hierarchical Genome Assembly Process (HGAP) version 3 was used for the assembly. Consequently, a single contig over 2.0 Mb was assembled. For the assembly polishing, the contig was mapped with 300-bp paired-end reads obtained by MiSeq sequencing (Illumina, San Diego, CA) using the CLC Genomic Workbench (Qiagen, Hilden, Germany). After the polishing, we obtained a single 2.1-Mb contig. To obtain the whole genome, a PCR primer set of the coding regions of the 5′ and 3′ nucleotide sequences of the contig was designed and confirmed by Sanger sequencing.

The complete genome sequence was 2,045,062 bp, with 39.9% G+C content. We observed 1,983 protein-coding regions, each with 4 copies of 5S, 16S, and 23S rRNA, and 58 tRNAs using DDBJ Fast Annotation and Submission Tool beta versions (6).

We believe these data will be used for molecular analysis of multidrug resistance and invasive mechanisms and epidemiological analysis of spreading *S. pneumoniae* 19F in future studies to prevent disease.

### Accession number(s).

This whole-genome sequence study has been deposited at DDBJ/ENA/GenBank under the accession number AP018391. The version described in this paper is the first version.
